# Co‐Designing AI‐Generated Vaping Awareness Materials With Adolescents and Young Adults: A Qualitative Study

**DOI:** 10.1111/dar.70022

**Published:** 2025-08-18

**Authors:** Tianze Sun, Gary Chung Kai Chan, Daniel Stjepanović, Tesfa Yimer, Giang Thu Vu, Carmen Lim, Caitlin McClure‐Thomas, Charlotte Russel, Jason Connor, Wayne Hall, Leanne Hides, David Hammond, Timo Dietrich, Daniel Erku, Benjamin Johnson, Janni Leung

**Affiliations:** ^1^ The National Centre for Youth Substance Use Research, School of Psychology The University of Queensland Brisbane Australia; ^2^ Discipline of Psychiatry, School of Medicine The University of Queensland Brisbane Australia; ^3^ School of Public Health Sciences University of Waterloo Waterloo Canada; ^4^ Social Marketing @ Griffith, Department of Marketing Griffith University Gold Coast Australia; ^5^ Menzies Health Institute Centre for Applied Health Economics Griffith University Brisbane Australia

**Keywords:** artificial intelligence, co‐design, health promotion, qualitative, vaping, young people

## Abstract

**Introduction:**

Developing mass meda campaigns to address rising youth vaping rates in Australia is timely and resource‐intensive. Generative AI offers scalable content production, but little is known about youth perceptions of AI‐generated multimedia materials or how their feedback can inform co‐design processes.

**Methods:**

We conducted a two‐phase qualitative study in Queensland, Australia. Phase 1 explored adolescent (*n* = 10, ages 13–20) responses to 120 vaping awareness materials produced using an automated‐AI framework. Focus group participants sorted materials into ‘effective’ and ‘ineffective’ piles and provided feedback. Based on feedback and quality criteria, 25 revised materials were created using an AI co‐design framework incorporating iterative, few‐shot prompting and manual text‐image integration. Phase 2 explored young adult (*n* = 9, ages 18–25) perceptions of revised materials via semi‐structured interviews. Inductive thematic analysis was conducted.

**Results:**

Phase 1 participants rejected automated‐AI‐generated materials due to misaligned text‐image combinations, artificial imagery, unrealistic vaping devices, and inauthentic language. Phase 2 identified six key characteristics of effective AI‐co‐designed materials that aligned with established health communication principles including visual appeal; focus on immediate consequences; relevance to youth; provision of practical advice; avoidance of ambiguity and fearmongering; and integration of multiple themes to reach diverse youth audiences.

**Discussion and Conclusions:**

AI tools can rapidly generate messages but an AI‐co‐design framework incorporating expert input and audience feedback is required to produce materials that are relevant, authentic, and evidence‐based. This framework offers a promising pathway for developing timely, scalable responses to public health challenges such as youth vaping; though continued research is needed for effective and ethical implementation across diverse contexts.

**Summary**

- Artificial Intelligence (AI)‐generated vaping awareness materials created using fully automated AI methods were rejected by youth due to poor visual alignment, artificial imagery, unrealistic vape depictions and inauthentic language.
- Participants identified characteristics of effective AI‐co‐designed materials that aligned with established health communication principles.
- A structured AI‐co‐design approach involving youth feedback and expert input successfully addressed most visual issues.
- Challenges with language authenticity persisted across both phases.
- AI, when paired with co‐design, offers a scalable, rapid solution for developing youth‐centred health campaigns aligned with evidence‐based communication principles.

## 1 Introduction

The high prevalence of youth vaping in Australia is a significant public health concern. Nationally representative data revealed that 17.9% of Australians aged 15–24 currently vape in 2022–23, representing a nearly four‐fold increase from 4.5% in 2019 [1]. Although vaping delivers fewer harmful toxins than combustible cigarettes, it still exposes users to harmful chemicals, including high levels of nicotine, respiratory irritants and carcinogens, which increase risks of nicotine dependence, respiratory damage and cardiovascular diseases, particularly when used from a young age [2].

For decades, Australia's comprehensive tobacco control strategy, including mass media campaigns, has effectively reduced smoking rates at the population level by shaping the public's attitudes, beliefs and behaviours around smoking [3]. Emerging research shows that vaping awareness mass media campaigns are similarly effective in shifting young people's attitudes and behaviours, especially when multi‐themed messaging approaches are used [4].

Despite this potential, most anti‐vaping campaigns in Australia only focus on health‐related consequences [5], missing out on other persuasive message themes like financial costs, industry manipulation, nicotine addiction, and social norms [6, 7]. This is due to the high costs and time demands associated with conducting in‐depth formative research, writing and designing materials, and pretesting them over extended periods [8, 9]. In Australia, public health advocates warned about increases in youth vaping in early 2018, but the first state mass media campaign launched only 4 years later, in 2022 [10]. Such delays severely hamper Australia's ability to respond to emerging health concerns and prevent community harm.

Generative Artificial Intelligence (AI), including Large Language Models (LLM) for text and Diffusion Models (DM) for images, offers a promising solution to these challenges. General purpose LLMs, such as OpenAI's GPT‐4 and Anthropic's Claude 3, can rapidly produce large volumes of human‐like text in response to user *prompts* (a text instruction). Popular DM platforms such as MidJourney and DALL‐E similarly respond to *prompts* to quickly produce high‐quality images, although their application in public health communication remains largely unexplored. Together, these tools enable the rapid creation of diverse multimedia materials across a wide range of public health topics [8]. This capability may be particularly valuable in low‐resource settings such as low‐ and middle‐income countries, where traditional campaign development is often constrained by limited infrastructure and funding, yet where public health challenges such as tobacco and vaping use are widespread [11].

Although recent studies have shown that AI‐generated text‐based health promotion messages can match or even exceed human‐written messages in clarity, persuasiveness, and quality [8, 12, 13], AI‐generated materials must be co‐designed with input from experts and intended audiences to be effective [14]. General‐purpose AI models, when used in fully automated ways, can produce outputs that are inaccurate, outdated, or inappropriate. Fully automated AI approaches such as ‘zero‐shot promptin’, where the AI is given no relevant examples to guide output [15] and *single‐prompt usage*, where the first AI‐generated output is accepted without iterative refinement, lead to several well‐documented issues [16]. These include factual inaccuracies or hallucinations [17], outdated information due to AI models' lack of real‐time updates [14, 18], and biased content that may exclude or misrepresent diverse groups, as current AI systems are primarily trained on vast internet‐sourced datasets that reflect English‐speaking, Western cultural norms [19]. In the context of youth‐targeted AI‐generated health communication, these risks can damage credibility, reduce engagement and ultimately undermine campaign effectiveness [16, 18].

To overcome these limitations, we propose an AI‐co‐design framework for youth health messaging. We define AI‐co‐design as a collaborative development process in which AI‐generated content is iteratively refined through structured input from target audiences and domain experts. This approach integrates iterative AI techniques such as ‘few‐shot prompting’, where developers provide AI with relevant examples to guide content generation, and *iterative prompting*, where outputs are revised over multiple rounds based on feedback from target users and domain experts to ensure outputs meet desired quality standards [8, 12, 13, 20].

Evidence supports the effectiveness of AI‐co‐design approaches. For example, AI‐generated vaping prevention messages created using few‐shot prompting were perceived by young adults (aged 18–24 years) as slightly more effective than human‐written ones [12]. Similarly, Karinshak et al. [14] found that GPT‐3 generated COVID‐19 pro‐vaccination messages developed through iterative refinement outperformed human‐written messages from the US Centers for Disease Control and Prevention in effectiveness and positive attitude formation.

**FIGURE 1:**
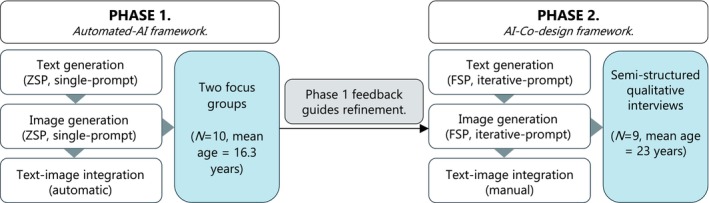
Two‐phase methodology for developing and evaluating AI‐generated vaping awareness materials. Phase 1 used an automated‐AI framework (ZSP; zero‐shot prompting, single prompt usage, automatic text‐image integration). Based on Phase 1 analysis, Phase 2 transitioned to an AI‐co‐design framework (FSP; few‐shot prompting, iterative prompting, manual text‐image integration).

Current studies on AI‐generated health promotion materials have primarily focused on text‐based messages generated through LLMs. Far less attention has been given to AI's potential to create multimedia materials combining images and text, despite visual content dominating social media platforms where young people are most active [21]. Additionally, most existing evaluations rely on quantitative ratings, which do not capture young people's deeper insights into why certain AI‐generated messages work or fail, and how to improve them. To address these gaps, this qualitative study explores youth perceptions of AI‐generated vaping awareness multimedia materials targeted at adolescents and young adults aged 13–25 years, and how their feedback can inform the iterative refinement of such materials through a co‐design process (Figure 1). Specifically, we aim to:

1. Phase 1: Explore reasons adolescents (aged 13–20 years) endorse or reject vaping awareness materials created with an automated‐AI framework through a pile‐sorting activity via focus groups.
2. Phase 2: Revise materials using an AI‐co‐design framework based on Phase 1 feedback and explore young adults (aged 18–25 years) perceptions of the revised material's relevance, appeal and potential effectiveness.

Together, these two phases address the practical question facing health communicators on whether novel AI tools can simply be used as standalone solutions (Phase 1) or require human integration (Phase 2) for effective health communication.

## 2 Phase 1—Focus Groups

This study followed the Consolidated Criteria for REporting Qualitative Research (COREQ) guidelines [22]. Ethics approval was obtained from The University of Queensland Human Research Ethics Committee (2024/HE000318).

### 2.1 Methods

#### 2.1.1 Material Development

In January 2024, we used ChatGPT‐4 for text and Midjourney v6 for images to develop 120 materials using an automated‐AI framework consisting of zero‐shot prompting (AI performs tasks without relevant examples) and single‐prompt usage (outputs used without iterative prompting) (see Data S1A for prompt used) [14, 15]. Generated text was then overlaid onto corresponding images using Canva's automatic bulk creation feature. Although this process was guided by J.L., who is a researcher with expertise in youth vaping and health communication, minimal domain‐specific input was provided to establish a baseline for understanding what kind of outputs AI would produce without substantial human guidance or iterative refinement.

#### 2.1.2 Sample Recruitment

Phase 1 employed purposive sampling through an established partnership with a Local Youth Action Group (LYAG), a community organisation led by the Logan City Council in Brisbane, Australia. This sampling strategy was selected to access adolescents (ages 13–20 years) who had prior exposure to youth‐focused health initiatives, as our team had previously collaborated with LYAG on school‐based vaping prevention programmes. We sought to recruit a small, diverse group (*n* = 10), consistent with established qualitative research guidelines suggesting that focus groups with 6–12 participants are appropriate for thematic analysis aimed at exploring perceptions and informing iterative material refinement [23]. The LYAG coordinator shared study information with youth members during regular meetings; interested members were invited to participate.

#### 2.1.3 Procedure

Five moderators (T.S., D.S., C.M.‐T., C.L., G.V.) were trained using Krueger [24] focus group facilitation guide. Sessions were conducted at the University of Queensland, St Lucia campus at participants' request to visit the university. The LYAG Community Development Officer was present throughout the sessions to ensure participants' comfort and maintain continuity with usual practice. Each session began with icebreakers, a vaping discussion and study overview. Participants then provided written informed consent and completed brief paper‐and‐pencil surveys on their sociodemographic characteristics and vaping status. A set of AI‐generated materials was shown, and participants were asked to sort them into: (i) effective; and (ii) ineffective at deterring youth vaping. Moderators used probing questions to explore why materials were selected as effective or ineffective and how effectiveness could be improved (standardised timeline in S2).

Given that Phase 1 was primarily focused on quality improvement to refine materials for Phase 2 and included young adolescents (ages 13–20), sessions were not audio‐recorded to maintain a comfortable, low‐pressure environment. Instead, multiple moderators took structured field notes of participants' reasoning, verbatim quotes, and non‐verbal cues to ensure robust data collection. At the session's end, moderators provided a summary of key points and invited participants to correct and add insights. To avoid introducing bias related to existing perceptions of AI, participants were told that the materials were AI‐generated at the end of the sessions. Previous research shows that AI disclosure can reduce message credibility and engagement [12, 25], so this ensured that participants evaluated the materials based on their perceived relevance and effectiveness, regardless of their source. Sessions lasted 120 min with two 10‐min breaks. Each participant received a $30 AUD gift card, lunch and travel reimbursement.

#### 2.1.4 Data Analysis

Post‐session, moderators debriefed to discuss observations and interpretations. Field notes and reflections from debriefs were compiled and imported into NVivo (Version 12) for coding and analysis. Given the data‐driven nature of the study, an inductive thematic analysis following Thomas' [26] approach to qualitative data was conducted. Two authors (T.Y., T.S.) independently conducted close readings. Meaningful text segments were identified and coded into three predetermined categories aligned with the study objectives: (i) endorsement reasons; (ii) rejection reasons; (iii) suggestions for improvement. Within each category, specific themes were developed inductively from the text. T.Y. and T.S. met regularly to compare their coding, discuss discrepancies and refine themes until consensus was reached.

### 2.2 Results

Participants in the two focus groups (*N* = 10) had a mean age of 16.3 years and comprised six females and four males (Table 1). Three participants (30%) reported prior vape use, seven (70%) had never vaped, and three spoke a language other than English at home.

**TABLE 1:** Participant demographic characteristics, *n* = 19.

Characteristic	Phase 1, *n* (%)	Phase 2, *n* (%)
Sample	10	9
Gender		
Male	6 (60.0%)	5 (55.6%)
Female	4 (40.0%)	4 (44.4%)
Vaping status		
Never used	7 (70.0%)	4 (44.4%)
I only tried them once or twice	1 (10.0%)	3 (33.3%)
At least weekly, but not daily	0 (0.0%)	2 (22.2%)
Daily	0 (0.0%)	0 (0.0%)
I used to use them, but no longer use	2 (20.0%)	0 (0.0%)
Language other than English		
Yes	3 (30.0%)	4 (44.4%)
Background (written)	Aboriginal Samoan Vietnamese‐Australian Teochew Chinese‐Australian Burmese‐Australian Caucasian‐Australian	Chinese‐Australian Korean‐Australian Caucasian Australian
Age (years), range, *M*	13–20, 16.3	18–25, 23

#### 2.2.1 Endorsement Reasons

Participants endorsed materials based on three key attributes (Table 2): visual appeal, clear communication style and ability to create personal resonance with youth.

**TABLE 2:** Key themes and illustrative quotes from thematic analysis of Phase 1 focus group data.

Overarching categorises	Themes	Quotes
Endorsement reasons	Visual appeal	‘I like the bright, contrasting colours’.
	Direct and clear messaging	‘The ones we endorsed were straightforward and to the point’.
	Personal resonance	‘You can see yourself being her’,
Rejection reasons	Misalignment between image & text	‘Text and image [were] not matched’ ‘Look like an ad about driving because image is not relevant’ (regarding Figure 2b)
	Artificial visual quality	‘It looks too perfect, like a stock‐like photo’
	Unrealistic device representation	‘[I've] never seen those kinds of vapes before. You need to put the ones actually used by teens to be effective’.
	Inauthentic language and message	‘Sounds like my parents’ ‘Wording is strange’
Ideas for improvement	Visual style diversity	‘Try different styles like cartoons, celebs, quotes and things’ ‘They all look the same, change the fonts and layout’
	Realistic device representation	‘Make it [the vape in photo] more like a puff bar’
	Substantive reasoning	‘Include some actual information or stats about harmfulness rather than just catchy sayings’

#### 2.2.2 Rejection Reasons

**FIGURE 2:**
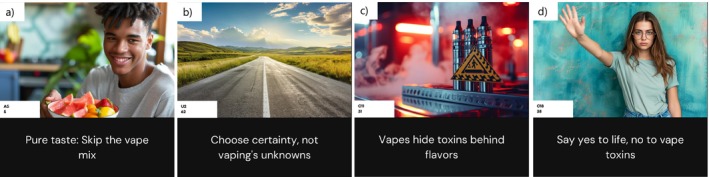
Examples of AI‐generated materials created with an automated‐AI framework (ZSP; zero‐shot prompting, single‐prompt usage, automatic text‐image integration).

Four main themes emerged as reasons for rejection (Table 2). The primary concern was poor image‐text alignment, particularly when generic images failed to support text (see Figure 2a). Other reasons included AI‐generated images appearing artificial, unrealistic representations of vaping devices that did not match products commonly used by youth (Figure 2c.) and inauthentic, awkward language. Simple messages like ‘just say no’ (Figure 2d) were criticised as alienating and described as ‘cringe’ or resembling ‘a middle‐aged woman's Facebook post’.

#### 2.2.3 Suggestions for Improvement

Participants offered three key suggestions for improving materials (Table 2): incorporating diverse visual styles, including realistic images of vaping devices, and providing more substantive information about reasons to not vape rather than relying on simple slogans.

## 3 Phase 2—Individual Semi‐Structured Interviews

### 3.1 Methods

#### 3.1.1 Material Refinement

The themes related to reasons for rejection, identified in Phase 1, could be attributed to the automated‐AI framework used, which had minimal human oversight and lacked iterative refinement. To address participants' feedback, we developed and implemented a structured AI‐co‐design framework for Phase 2 that incorporated targeted youth feedback within a systematic iterative process [8, 14].

Our AI‐co‐design framework consisted of four key steps: (i) structured few‐shot prompting with relevant examples; (ii) iterative text refinement; (iii) visual content generation; and (iv) manual integration of text and images using Canva. Each step was evaluated against established quality criteria: accuracy, relevance, and persuasion attempt [14] and Phase 1 feedback (see Table S3 for definitions). This was guided by a researcher (T.S.) with domain expertise, relevant lived experience, and as someone within the target age group (25 years) at the time of material development. This process is described in detail in Data S1B, which includes examples of prompt refinement, image generation logic, and integration workflows.

In May 2024, a new set of materials was created using Claude‐3 Opus for text and Midjourney v6 for images. We transitioned from GPT‐4 used in Phase 1 to Claude‐3 Opus in Phase 2 based on emerging research showing Claude's superior readability and reliability performance in generating health‐related content [27] and stronger ethical safeguards against producing misleading health information [28]. A total of 25 refined materials were created for each of the following five themes: nicotine addiction, health impacts, financial impacts, industry manipulation, and social norms. All materials can be found in the study GitHub repository (https://github.com/gckc123/AIvaping).

#### 3.1.2 Sample Recruitment

Young adults aged 18–25 years were recruited using convenience sampling via university advertisements and snowball sampling through our research centre's student networks in Queensland, Australia. This shift in sampling ensured that perspectives on AI‐co‐designed materials were not influenced by previous experiences with the automated‐AI materials or research process.

#### 3.1.3 Procedure

Individual, semi‐structured interviews were conducted between June and July 2024, either in private rooms at The University of Queensland or via a secure video conferencing platform, depending on participant preference. All participants provided verbal consent following a discussion with the interviewer (G.C.) regarding the study purpose. A semi‐structured interview guide S4 developed by the research team and refined through pilot testing with undergraduate students was used.

Interviews were audio and video recorded and lasted 28 min on average. Participants evaluated 5 AI‐generated materials at a time, providing feedback on each material's relevance, appeal, and potential effectiveness. Similar to Phase 1, the AI‐generated nature of the materials was disclosed at the end of the interviews. Participants received a $30 AUD gift card.

#### 3.1.4 Data Analysis

All interviews were transcribed via Otter.ai software, anonymised and imported into NVivo. An inductive thematic analysis was conducted, given the study's data‐driven nature [26]. The first author (T.S.) reviewed the video recordings and transcripts, identifying and coding text segments related to the aim (characteristics of effective youth vaping awareness materials). Through multiple readings, T.S. developed an initial set of over 50 categories describing material characteristics (e.g., clear fonts, youth language). These descriptive categories were subsequently refined and combined into broader concepts through multiple team discussions (B.J., G.C., T.S., T.Y., G.C.) until consensus was reached on the final themes. For validation, T.S. conducted stakeholder checks by presenting preliminary themes to a Phase 2 participant. The final themes were documented with clear labels, descriptions and illustrative quotes.

### 3.2 Results

Participants (*n* = 9) had a mean age of 23 years, with five females and four males (Table 1). Five participants (56%) reported vaping experience; four (44%) had never vaped, and four spoke a language other than English at home. Inductive thematic analysis revealed six key themes related to characteristics of effective youth vaping awareness materials.

#### 3.2.1 Theme 1: Capture Attention and Engagement Through Visual Appeal

Participants emphasised the importance of visual appeal in capturing initial interest, particularly on social media platforms, where users rapidly scroll through content. Effective materials featured bright colours, sharp contrasts, clear typography, and vivid imagery.

‘My attention span [was] captured by colours which would stand out in [an] Instagram feed.’(Female, 24)

**FIGURE 3:**
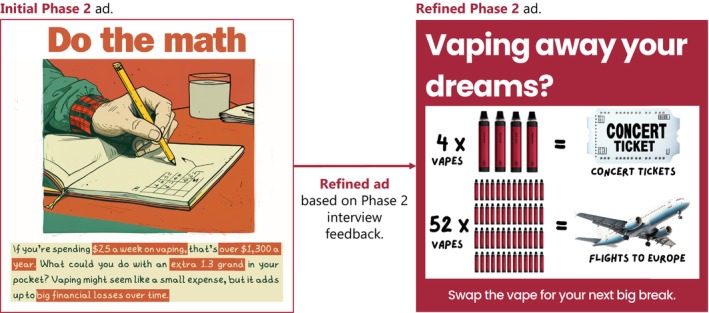
Evolution of an ad using financial impact messaging theme, refined based on Phase 2 interview feedback. Demonstrates the transition from a purely text‐based description ($25 a week) to concrete visual comparisons that appeal to young people's interests (concert tickets and flights to Europe).

Concrete visual comparisons made messages more impactful and easier to understand. For example, participants favoured visual analogies that compared vaping harms to familiar household chemicals over purely text‐based presentations. Similarly, participants suggested that visually framing the monetary cost of vaping in terms of relatable alternatives, such as concerts or trips, could make messages more persuasive (Figure 3):

‘If you were to show that 25 vapes equals one concert or a trip, it would give it more value in someone's head.’(Female, 23)

#### 3.2.2 Theme 2: Focus on Immediate Consequences Over Long‐Term Risks

**FIGURE 4:**
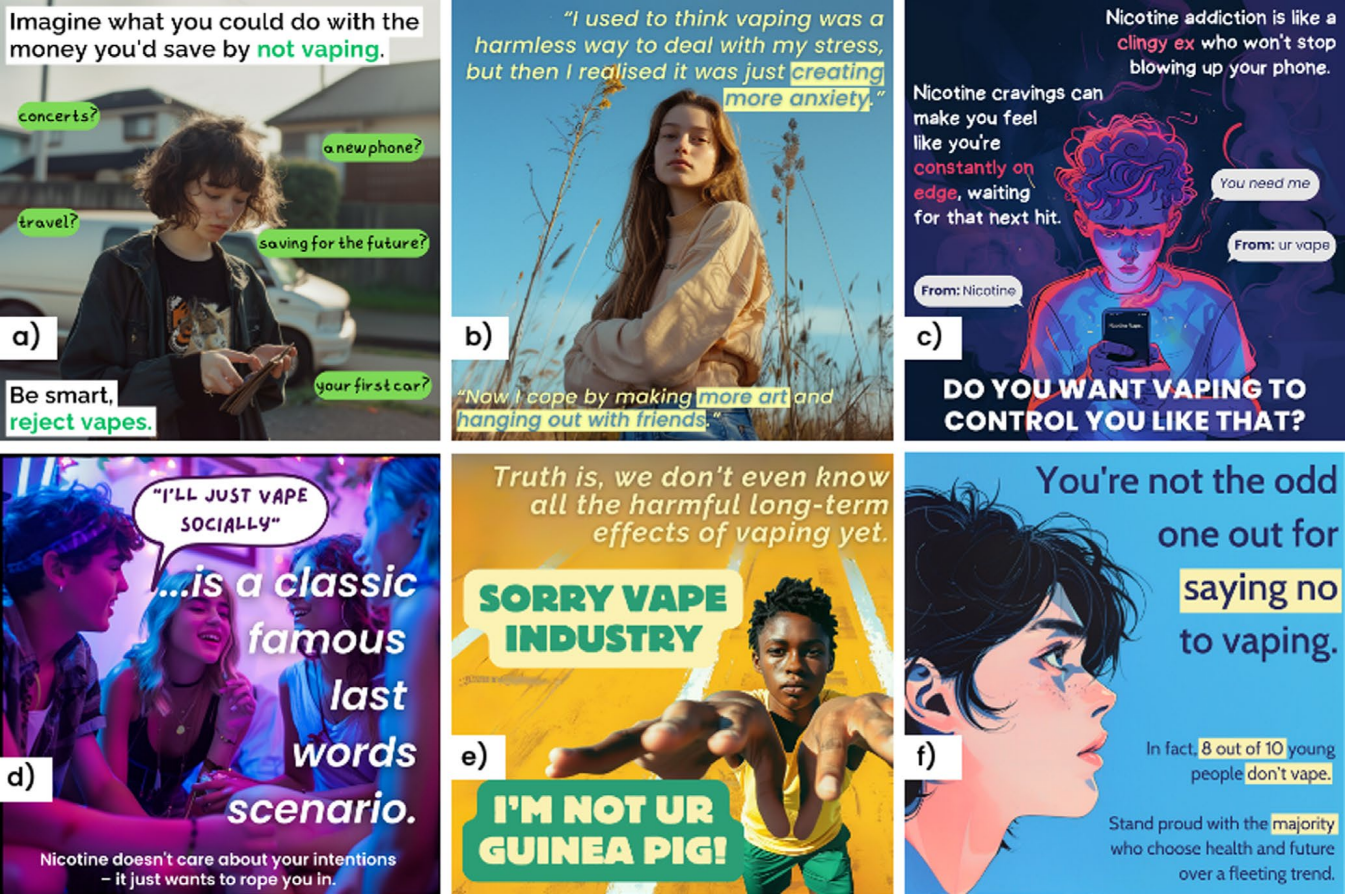
Examples of new AI‐generated materials used in Phase 2 semi‐structured interviews. Created using AI‐co‐design approach (few‐shot prompting, iterative prompting, and manual text‐image integration).

Participants emphasised the importance of focusing on immediate and tangible outcomes rather than distant or abstract risks (Figure 4a). Long‐term outcomes, whether related to health or financial impacts, often seemed too remote to influence behaviour and sometimes even triggered anxiety about future planning.

‘As awful as it sounds, not a whole lot of people my age are concerned about their health. We're young, we're 18 or 20, and we're just living life.’(Female, 18)

#### 3.2.3 Theme 3: Make Messages Relevant and Relatable to Youth

The perceived effectiveness of materials depended on their ability to reflect familiar language, imagery, and scenarios from participants' everyday lives. References to youth culture, such as ‘clingy e’ and ‘red flag’ resonated with some (Figure 4c). However, others cautioned against the overuse of youth slang and warned that attempts to mimic their language too closely felt disingenuous.

‘I feel like maybe it's just trying a little too hard to communicate with [young people]. The jargon, spelling of your being shortened to “ur” and uses of, like, clingy ex and that stuff. It might come across as disingenuous, like, oh, this is made by someone older and they're clearly trying to reach me.’(Male, 24)

Similarly, imagery, such as physical wallets, was outdated in an era dominated by digital payments.

‘The graphic … what is it? Oh, it's a wallet! But we all use Apple Pay now, and so I'm like, “What's a wallet again?” It took me ages to figure out what he was actually holding. I like the message, but the graphic—I didn't make that link.’(Female, 18)

Participants responded positively to materials that depicted familiar and realistic scenarios. An ad featuring the phrase ‘I'll just vape socially’ (Figure 4d) resonated with all participants because it mirrored conversations they had observed or participated in with peers. This message effectively captured the gradual shift from casual use to dependence, which participants described as relatable and thought‐provoking.

‘I'll just vape socially … I've heard that a lot. So, if someone has heard that and then they hear this, this is quite a powerful statement. It's a scene that everyone could relate to.’(Female, 23)

#### 3.2.4 Theme 4: Provide Clear Calls to Action Using Supportive Language

Participants responded positively to materials that provided practical strategies for behaviour change. Materials that emphasised coping strategies, personal growth, and the importance of seeking help were seen as inspiring and effective (Figure 4b).

‘I like this. It's showing that there's other ways to deal with your stress and anxiety, like talking about it or exercising or doing something that you want to do.’(Female, 22)

‘Good that there is something that's hopeful for even long‐time vapers to think, “Oh, there's still a way for me to get out of the addiction”.’(Male, 22)

However, participants cautioned against materials that suggested pride in not vaping or implied judgement of those who do. Such messages were perceived as abrasive and alienating, reinforcing the importance of a supportive, non‐judgmental tone that encourages positive change without demonising individuals who vape.

#### 3.2.5 Theme 5: Avoid Ambiguity and Fearmongering in Vaping Risk Communication

Participants expressed frustration with materials that acknowledged uncertainty about the long‐term risks of vaping (Figure 4e) or used hedging language like ‘may’ or ‘migh’. Participants said that such ambiguity left room for interpretation, downplayed potential harms, and justified continued use for some.

‘I don't know why the message just self‐admittedly doesn't know all the long‐term effects. It's almost giving leeway to the opposite argument of, “we don't know all the long‐term effects, so maybe they're not that bad”.’(Male, 23)

However, presenting definitive claims about vaping harms also undermined credibility and trust, as some participants perceived these as exaggerated and fearmongering. Such tactics discouraged engagement and created doubt about the reliability of the source.

‘It's not like I'm buying it off the street … they're mass‐manufactured. I imagine they go through a pretty thorough process of quality checking. So, if I saw these, I don't know how much I'd believe. I feel like they're fearmongering, almost.’(Male, 24)

‘It's a bit much. [That ad] sounds like every puff is going to kill you. It just doesn't match what I know.’(Female, 25)

#### 3.2.6 Theme 6: Tailor Messages for Diverse Values and Motivations

Participants' responses revealed how individual differences fundamentally shape message effectiveness. The same materials often elicited opposing reactions, highlighting why one‐size‐fits‐all messaging approaches may fail to resonate across diverse youth audiences. For example, the financial cost of vaping resonated strongly with participants who were concerned about money:

‘The money one is one of the biggest. I don't know anybody in any of my spheres who's not worried about money. We're all like, “Oh, that's too much money. Oh, we've got to pay rent, uni fees or car, petrol, and rego”.’(Female, 18)

Although others felt that it was less compelling and believed that enjoyment and relaxation should be prioritised over saving mone;

‘I don't think young people care a lot about how they spend their money.’(Male, 22)

Similarly, there were divergent responses to the message that the majority of young people do not vape (Figure 4*f*). Some participants appreciated the sense of belonging and social acceptance this messaging offered, particularly for non‐users:

‘“Over 80% don't vape”, I like this because it doesn't feel like I'm the only one [not vaping]. It feels like the other way around.’(Female, 25)

Others found such messaging less appealing, arguing that nonconformity was a value embraced by youth:

‘There's a lot of young people that maybe find an appeal in being away from the mainstream.’(Male, 24)

These contrasting responses demonstrate how personal values (such as financial responsibility versus immediate gratification) and identity factors (conformity versus individuality) critically influence message reception. To address this variability, participants recommended combining multiple message themes to increase the persuasiveness of materials.

## 4 Discussion

This two‐phase qualitative study explored adolescent perspectives of automated‐AI‐generated youth vaping awareness materials and young adult perspectives of AI‐co‐designed vaping awareness materials. Although automated AI approaches proved efficient in quickly producing large volumes of content, Phase 1 highlighted challenges specific to visual and multimodal content generation including misaligned text‐image combinations, artificial imagery and unrealistic vaping device representations. These concerns speak to limitations in DM and the challenges of automated text‐image pairing. Inauthentic or awkward language was also a consistent reason for rejection, aligning with previous concerns about AI‐generated text‐based health messages [12, 13, 14].

Phase 2 introduced an AI‐co‐design framework featuring few‐shot prompting, iterative refinement and manual text‐image integration. This approach enabled us to address concerns raised in Phase 1. For example, Phase 2 participants did not raise concerns about text‐image misalignment, artificial visuals, or unrealistic vaping device depictions, which were predominant issues in Phase 1. However, concerns about language authenticity persisted, as captured in Theme 3 (relevant and relatable messages), suggesting that creating authentic youth‐oriented messaging may require direct involvement from the target audience regardless of AI capabilities. These observations, however, should not be interpreted as direct comparisons, as our study employed different age groups, methodologies and analytic approaches for the distinct research aims of each phase.

Participants emphasised that effective materials were visually appealing, focused on immediate consequences, relevant to youth, offered supportive and practical advice about avoiding or quitting vaping, avoided ambiguity and fearmongering in vaping risk communication, and integrated multiple themes to reach youth with different values and experiences. These themes align with established principles from decades of anti‐tobacco media campaign research [3] and behaviour change theories [29, 30, 31, 32], suggesting that AI‐generated content, when properly co‐designed, can reflect evidence‐based health communication standards.

### 4.1 Implications and Guidelines for AI‐Co‐Design in Public Health Campaigns

Throughout the co‐design process, we identified several advantages of AI that overcome longstanding barriers to producing timely, relevant, and engaging public health materials. First, AI is a promising tool for reducing the long timelines associated with iterative message refinement due to its rapid content generation and responsiveness to revising outputs based on audience feedback. Second, AI allowed for the simultaneous development of multiple message themes and can be further used to tailor materials to diverse youth subgroups with different identities, values, and experiences. Finally, AI facilitated novel and creative message‐image pairings that might not have emerged through traditional material development.

These benefits are realised when paired with structured guidance, expert input, and continuous audience validation. For health professionals considering AI co‐design for health messaging, we recommend the following: (i) define quality standards before generation begins; (ii) engage domain experts in prompt design and output evaluation; (iii) implement feedback loops with target audiences; and (iv) manually integrate text and visuals until AI capabilities improve.

### 4.2 Limitations and Future Directions

Several limitations should be considered when interpreting our findings. The use of different age groups, recruitment contexts, data collection methods, and analytic approaches across the two phases limits direct comparison of participant responses and affects the transferability of findings. The absence of audio recordings in Phase 1 reduced transcription accuracy and limited the depth of our thematic analysis, despite the use of structured field notes to mitigate this constraint. Focus group dynamics in Phase 1 may have introduced social desirability bias; however, this was partially offset by the use of individual interviews in Phase 2, which allowed for more in‐depth responses. Moreover, our small sample sizes, whereas aligned with established qualitative research guidelines [23], limit the breadth of perspectives captured. Although the shift from GPT‐4 (Phase 1) to Claude Opus 3 (Phase 2) may have introduced subtle differences in tone or quality, these variations were likely minimised by our iterative co‐design approach, which ensured that the final materials reflected Phase 1 feedback and met quality standards, regardless of the LLM model used. Finally, not measuring smoking status limited our understanding of how these materials might influence individuals who could benefit from switching to e‐cigarettes for smoking cessation.

Despite these limitations, our study provided novel findings with implications for the integration of AI technologies in health communication and youth‐targeted public health interventions. Although the individual design principles that emerged align with established health communication literature, our comparison of automated AI versus AI‐co‐designed approaches represents an empirical examination of how young people respond to different AI‐generated health materials. Our findings demonstrate that using AI's automated function in a simple way resulted in inferior results due to text‐image misalignment, artificial imagery, and inauthentic language. These AI‐specific challenges that differ fundamentally from traditional campaign development issues need to be taken into account with the increasing uptake of AI use in healthcare settings. Our study suggests that while AI cannot fully automate effective health communication, it can significantly enhance efficiency and scalability when implemented through co‐design approaches that incorporate expert input and audience feedback. This has immediate implications for public health organisations increasingly adopting AI technologies, providing actionable guidance on when and how these tools can be effectively integrated while maintaining message authenticity and effectiveness.

Given that e‐cigarettes present both risks for youth [2] and potential benefits as cessation tools for adults who smoke [33], future studies should explore how perceptions differ by smoking status. This research is needed for developing targeted messaging that avoids unintended consequences for harm reduction efforts. Studies should also examine how disclosing the AI‐generated nature of materials affects their perceived credibility and effectiveness [12, 25]. As AI technology evolves, research exploring different approaches to text‐image integration will be valuable. Most importantly, longitudinal studies measuring actual behavioural outcomes will be essential to understand the sustained impact of AI‐generated campaigns.

## 5 Conclusion

This study contributes to a growing body of work examining how generative AI can be used to support the development of youth‐focused health promotion materials. Although AI tools are capable of rapidly producing persuasive text and engaging visuals, our findings emphasise that an AI‐co‐design framework incorporating expert input and audience feedback is required to produce materials that are relevant, authentic and evidence‐based. The value of AI in this context lies not in replacing human judgement, but in enabling the rapid creation and iterative refinement of diverse message sets. For practitioners, particularly those working within resource‐limited settings, AI offers a promising and practical tool, one that, if integrated thoughtfully, can support the creation of timely, targeted and scalable health campaigns that speak to the varied experiences, identities, and motivations of young people.

## Author Contributions

**Tianze Sun:** conceptualisation, methodology, validation, formal analysis, investigation, resources, data curation, writing – original draft, writing – review and editing, visualisation. **Gary Chung Kai Chan:** methodology, validation, investigation, data curation, writing – review and editing, supervision, project administration, funding acquisition. **Daniel Stjepanović:** methodology, validation, investigation, data curation, writing – review and editing, supervision, project administration. **Tesfa Yimer:** validation, writing – review and editing, formal analysis, visualisation. **Giang Thu Vu:** investigation, writing – review and editing. **Carmen Lim:** investigation, writing – review and editing. **Caitlin McClure‐Thomas:** investigation, writing – review and editing. **Charlotte Russel:** investigation. **Jason Connor:** writing – review and editing. **Wayne Hall:** writing – review and editing. **Leanne Hides:** writing – review and editing. **David Hammond:** writing – review and editing. **Timo Dietrich:** writing – review and editing. **Daniel Erku:** writing – review and editing. **Benjamin Johnson:** formal analysis, writing – review and editing. **Janni Leung:** resources, writing – review and editing.

## Conflicts of Interest

D.H. has provided paid expert witness testimony on behalf of public health authorities in response to legal challenges from tobacco, vaping, and cannabis companies.

## Supporting information


**Data S1:** dar70022‐sup‐0001‐supinfo.

## Data Availability

The data that support the findings of this study are available on request from the corresponding author. The data are not publicly available due to privacy or ethical restrictions.
