# Supplementary material for: Co‐Designing AI‐Generated Vaping Awareness Materials With Adolescents and Young Adults: A Qualitative Study
Source: Drug Alcohol Rev. 2025 Aug 18;45(1):e70022. doi: 10.1111/dar.70022 (PMC12680904; doi:10.1111/dar.70022)
Supplement: Supplementary file 1 — Data S1: dar70022‐sup‐0001‐supinfo. [file DAR-45-0-s001.docx]

# SUPPORTING INFORMATION

## S1A. Zero-shot prompt used for ChatGPT in Phase 1

| User prompt: *You are representing a professional health promotion and research institute, and you would like to create health promotional content to post on Instagram to deter youth vaping. Create a list of 20 messages to deter vaping, targeted at young people aged 13-25 years, with a focus on the theme that <theme description>. Make 10 of them positive (i.e. highlighting the benefits of not vaping), and 10 of them negative (i.e. highlight the harms or drawbacks of vaping). The messages should be about 80 characters.*  *First, please make all 20 messages related to <theme description>.*  *Second, suggest a relevant image to be included for each message to enhance visual appeal to youth.*  *Third, provide a short descriptive MidJourney V6 image prompt for the images you suggested. The prompts should be descriptive, describing what is included in the image, not describing what is to be excluded in the image, and suitable for a teenage to young adult audience.*  *Fourth, create a table with four columns for the message number and for the output for the three steps above.*  *Finally, separate from the table, please also concatenate all the MidJourney V6 prompts into one sentence, without quotation marks, separated by “;”* |
| --- |

## S1B. Material refinement through AI-co-design approach in Phase 2

## STEP 1: Structured few-shot prompting method for Claude

The structured prompt format consisted of four key components: (1) positioning the AI as a health promotion expert, (2) defining message theme to provide context and background, (3) an example section that demonstrates effective messaging through existing campaign materials by established health agencies, and (4) detailed instructions for generating engaging, evidence-based messages that resonate with youth aged 13-25. Below is an example prompt used for text generation. This structured prompt format can be easily adapted for any vaping-related theme by replacing the [THEME] placeholder (e.g., financial impact, health consequences, social norms).

| User prompt: *You are an expert in youth health promotion and education. Your job is to create health promotional messages that will effectively dissuade youth from starting or continuing vaping. Many types of messages can deter youth. We will look at [THEME]. Here is a definition related to the [THEME] of vaping to help you create effective messages.*  *[Insert a clear and concise definition of the chosen theme, explaining its relevance to vaping prevention and how it can influence youth behaviour. This section should provide the necessary context for generating targeted messages.]*  *Here are some examples of vaping prevention messages from existing campaigns that focus on the [THEME] of vaping, to provide inspiration:*  *[Provide a few examples of existing vaping prevention messages that align with the chosen theme. These examples serve as inspiration and demonstrate effective messaging strategies.]*  *Carefully review the provided [THEME] of vaping definition and example messages. Then, brainstorm 10 unique, impactful and effective vaping prevention messages targeted at youth aged 14-25. The messages should focus on educating about and emphasising the [THEME] of vaping. When crafting your messages, keep the following in mind:*   - *Use language, tone and examples that will be relatable and resonant for the youth demographic.* - *Support your points with concrete facts and evidence.*   *Please generate 10 messages on the [THEME] of vaping now.* |
| --- |

**STEP 2: Iterative prompting to meet quality criteria and incorporate Phase 1 feedback**

For each theme, Claude-3 Opus initially generated 10 vaping awareness messages. We then evaluated each message against the three established quality criteria:

- Accuracy: Content must be factually correct and aligned with current scientific evidence
- Relevance: Content must be appropriate for and resonate with youth aged 13-25
- Persuasion attempt: Content must include a clear call to action or persuasive element

As well as specific feedback themes from Phase 1 adolescents: e.g.,

- Avoiding inauthentic language
- Including substantive reasoning rather than simplistic slogans like "just say no"
- Using direct, clear messaging that creates personal resonance

The 10 initial messages were reviewed, and those meeting both quality criteria and addressing Phase 1 feedback were retained. For messages that showed potential but had issues, we used targeted feedback prompts. Here is an example where we addressed the factual inaccuracy identified in initial review:

| Claude (from initial set of 10 messages): *"Vaping can lead to popcorn lung. Is that a risk you're willing to take?"*  User prompt (based on accuracy quality criterion): *"The 2nd statement contains inaccurate information. The EVALI outbreak (popcorn lungs) was caused by vitamin E acetate from illegal cannabis vapes, not regular nicotine vapes. Please revise messages to focus on established health risks of nicotine vaping with accurate information."* |
| --- |

Here is an example where we enhanced relevance based on combined criteria:

| Claude (from initial set of 10 messages): *"Addiction is like a clingy ex who won't stop blowing up your phone. Nicotine cravings can make you feel like you're constantly on edge, waiting for that next hit. Is that the kind of relationship you want with your vape?"*  User prompt (based on quality criteria and Phase 1 feedback on creating personal resonance): *"This message uses relatable language and creates personal resonance, which Phase 1 participants identified as effective. However, the question may suggest to young people that their relationship to a vape could be positive. Rephrase the question to avoid this implication."*  Claude (revised output): *"Sure, let's rephrase the question. Here's the edited version: Addiction is like a clingy ex who won't stop blowing up your phone. Nicotine cravings can make you feel like you're constantly on edge, waiting for that next hit. Don't let your vape control you like that."* |
| --- |

After multiple iterations, we selected the 5 strongest messages per theme (total 25 messages across 5 themes) that best incorporated both the quality criteria and feedback from Phase 1 adolescents. This selective and iterative approach ensured that our final messages directly addressed the concerns raised in Phase 1 while maintaining factual accuracy and persuasive appeal.

**STEP 3: Visual content generation**

After finalising the text message through iterative refinement, we used Claude-3 Opus to generate visual concepts matching each message. Here is an example of the prompt we used:

| User prompt: *"Provide some ideas for what image would best accompany this message: 'Addiction is like a clingy ex who won't stop blowing up your phone...'"* |
| --- |

The most appropriate visual concept was selected and used it to create a Midjourney v6 prompt. Here is an example user prompt that would be used for Midjourney to generate 4 images.

| User prompt: *"An illustration of a frustrated young person looking at their phone with multiple notifications, subtle warm lighting, no text overlay --ar 1:1 --v6”* |
| --- |

If outputs failed to meet quality standards, we iteratively prompted the model with guidance such as:

| User prompt: *“MidJourney is not great at generating realistic visuals of vapes. Update the prompts so that vapes don’t have to be included in them.”* |
| --- |

**STEP 4: Manually integrate text and images using Canva**

TS (with domain expertise, relevant lived experience, and as someone within the target age group (25 years) at the time of material development) manually integrated text and images using Canva and ensured materials were in line with quality standards and Phase 1 feedback (e.g., proper alignment, readability, and visual appeal).

## S2. Phase 1 standardised timeline

| **#** | **Task** | **Mins** |
| --- | --- | --- |
| 1 | **Start a conversation as soon as they come through the door.**   - *E.g., How was your Easter weekend?* | 2 |
| 2 | **Name badges**  (Moderators, assistant moderates, and participants put on name badge. Participants seated)   - *E.g., We have name badges for everyone today. Please write your name on it. These name badges help me remember your names, but they can also help you remember ours.* | 2 |
| 3 | **Participant consent and information forms**  (Assistant moderators to hand participants the set of forms below along with a pen)  Participant consent form  Participant information sheet (for them to keep)  Participant demographics form  Participant vaping status form (to be placed in separate pile/box) | 2 |
| 4 | **Introducing everyone**  (Moderator to introduce the assistant moderators and NCYSUR lab very quickly. Moderator to ask LYAG to introduce themselves around the room)   - *Could everyone please tell us your name, and:* - *E.g., how long you’ve been involved with LYAG?* - *E.g., what is your experience with youth vaping awareness and prevention workshops and what were your impressions?* | 4 |
| 5 | **Conversation about why it’s important to prevent vaping**   - *What are some of the negative health effects?* - *Do you know much about how the industry targets young people?* - *What are the environmental costs of vaping?* | 5 |
| 6 | **Vaping awareness materials & ground rules**  (Moderator to go through the types of materials they will see today and what they will need to do). Some grounds rules that the moderator will share include:   - *Don’t feel like you must respond to me all the time.* - *If you want to follow up on something that someone has said, you want to agree, or disagree, or give an example, feel free to do that.* - *Feel free to have a conversation with one another about these questions.* - *I am here to ask questions, listen, and make sure everyone has a chance to share.* - *There are no wrong answers. We expect that you will have differing points of view. Please share your point of view even if it differs from what others have said.* - *From past experience in groups like this, we know that some people talk a lot, and some people don’t say much. We really want to hear from all of you because you’ve had different experiences. So if you are talking a lot, I may interrupt you, and if you aren’t saying much, I may call on you. If I do, please don’t be offended. We have a lot to cover here today, and it’s just my way of making sure we get through all the questions and that everyone has a chance to talk.* | 3 |
| 7 | **Theme 1**  (Assistant moderators to hand participants the first pile of printed materials)   - *Here is the first set of 10 vaping prevention materials. These may be posted on Instagram.* - *Look through these materials and sort the materials into two categories. One category for materials that you think would be useful to help young people to avoid vaping. The second category is materials that you think wouldn’t be useful at helping young people to avoid vaping.* - *Think about what you like and what you don’t like about them. In 5 minutes, I will ask for your reactions.* | 5 |
| 8 | **Getting qualitative feedback**   - *Now what are your reactions to the first set of materials?* - *What’s your reaction to how it looks?* - *What’s your reaction to the content?* | 3 |
| 9 | **Prompting further feedback**   - *What would make the materials better, so you would be more likely to find it convincing?* - *Could you please share what it was about these materials that made you put them in the yes pile?* - *Why did these materials get put in the no category?* - *What did you think of the materials in this pile?”* - *What do you like best/least about this pile?* - *Which features of this pile do you particularly like?* - *What made you choose this ad to be in the yes pile?* | 2 |
| 10 | **Theme 2.**  (Assistant moderators to hand participants the second pile of materials)   - *Now we are going to look at another set of vaping prevention materials. Take a few minutes to look through these materials.* - *Imagine that you saw these materials on your Instagram feed. Which materials do you believe would prevent you from vaping and which materials would be less effective at preventing you from vaping?* - *As a group, sort these out into two piles. We can call one pile “yes, helpful at preventing me from vaping” and “no, not helpful in preventing me from vaping.”* - *Again, think about what you like and don’t like about these materials. In a few minutes, I will ask for your reactions.*   (Moderator to go back to #8 and #9) | 10 |
| 11 | **Theme 2, negative frame** (Moderators will do the same as #10, #8 and #9) | 10 |
| 12 | **Theme 2, positive frame** (Moderators will do the same as #10, #8 and #9) | 10 |
| 13 | **Break time! Snacks and light refreshments.**   - *Please help yourself to the snacks during our first break. We will come back in 5 minutes to work on the next set of materials.* | 5 |
| 23 | **Explaining the use of AI and summarising main discussion points**  (Assistant moderators to summarise the main discussion points and invites comments, amendments, or corrections)   - *Do you think we’ve missed anything in the discussion?* - *Is there anything we should have talked about but didn’t?* - *Suppose you had one minute to talk to the people who create vaping prevention materials, what would you say?* | 5 |
| 24 | **Concluding the focus group session.**  (Assistant moderators to provide participants with their $30 gift card)   - Thank group for participating. | 5 |
| 25 | **Lunch after the focus group**  (Moderators and assistant moderators take participants to St Lucy’s) | 60 |

## S3. Content coding definitions

| **Dimension** | **Description** |
| --- | --- |
| **Accurate** | Whether factual claims about vaping are supported by current scientific evidence and public health consensus. If message contains no factual claims (e.g., expresses opinion or emotion), coded as accurate by default. |
| **Relevant** | Whether the message specifically aims to deter young people from vaping. |
| **Attempts persuasion** | Whether a reasonable person would understand that the message is attempting to discourage youth vaping through clear communication of risks, consequences, or protective factors. |

## S4. Phase 2 semi-structured interview guide

*Introduction*

“Thank you for participating in our research project today. We’re developing a campaign aimed to decrease vaping among young people. We're working to understand how young people view and respond to anti-vaping campaign materials. As someone in our target age group, your perspective is extremely valuable to us.

I'll be showing you some materials we've designed for dissemination on social media and would like your honest feedback on whether they are appealing and relevant to young people like yourself. Please be completely candid - if you think something doesn't work or could be improved, we really want to know. Your input will help ensure our campaign resonates with young people. The session will take about 30-45 minutes. I'll show you different sets of materials and ask for your thoughts on each one.”

*Consent and confidentiality*

- Obtain verbal consent for participation and audio/video recording.
- Explain confidentiality measures.
- Remind participant they can skip any question or stop at any time.

*Main discussion*

Present 5 materials from each theme (Health/Financial/Social/Industry/Addiction).

*Initial reactions*

- "Take a few minutes to look through these materials. What are your initial thoughts?"
- "Which ones immediately catch your eye, and why?"

*Message effectiveness*

- "Which messages resonate most strongly with you? What makes them effective?"
- "Which ones do you think would be least effective? Why?"
- "How well do these messages connect with people your age?"

*Suggestions for improvement*

- "How could any of these be improved to better reach young people?"
- "Is there anything missing that you think should be included?"
- "Do you have any other suggestions for improving these materials overall?"

*Probing questions*

- "Can you tell me more about that?"
- "What makes you say that?"
- "Could you give me an example?"
- "How do you think other young people might react to this?"
- "What specifically about [element] stands out to you?"

*Conclusion*

- "Before we finish, is there anything else you'd like to share about these materials?"
- “Is there anything important we haven’t discussed?”
- Thank participants and provide participant compensation.
